# OA14 Sarcoidosis, Steroids and Saprophytes

**DOI:** 10.1093/rap/rkae117.014

**Published:** 2024-11-05

**Authors:** Kathryn Biddle, Jessica Chan, Thomas Juniper, Thomas Beaton, Keat Choong, Geraldine O’Hara, Michelle Fernando

**Affiliations:** Guys and St Thomas’ Hospital, London, United Kingdom; Guys and St Thomas’ Hospital, London, United Kingdom; Guys and St Thomas’ Hospital, London, United Kingdom; Sunshine Coast University Hospital, Birtinya, Australia; Sunshine Coast University Hospital, Birtinya, Australia; Guys and St Thomas’ Hospital, London, United Kingdom; Guys and St Thomas’ Hospital, London, United Kingdom

## Abstract

**Introduction:**

Infection remains a leading cause of morbidity and mortality in patients with systemic rheumatic diseases. Immunosuppressive therapies used to treat these conditions predispose to opportunistic infection, including Nocardia. Here we present a case of disseminated Nocardiosis in a patient with sarcoidosis, treated with oral corticosteroids and methotrexate.

**Case description:**

A 72-year-old Australian gentleman of European ancestry was diagnosed with multisystem sarcoidosis. At diagnosis, PET-CT demonstrated significant FDG-uptake in the parotid glands, spleen and pancreas with non-caseating granulomas observed on bone marrow and pancreatic biopsies. He was treated empirically for sarcoidosis with oral methotrexate and high dose oral prednisolone.

Two months later, he travelled to London. On the flight, he developed painful limb swellings and generalised weakness. Shortly after landing, he was admitted to a tertiary hospital.

On examination, he was febrile and transiently confused. There were tender, erythematous subcutaneous swellings affecting his right forearm, upper arm and calf.

Initial investigations demonstrated anaemia (Hb 98 g/L), leucocytosis (WCC 12.8*10^9^/L) and a raised C-reactive protein (77 mg/L). Co-amoxiclav was commenced for a presumed respiratory infection. CT thorax showed left apical subpleural consolidation with a cavitating lesion. CT head demonstrated two intracranial subcortical hypo-attenuating foci within the frontal and parietal lobes. After infection team review, ceftriaxone and metronidazole were commenced to ensure coverage for brain abscesses. Co-amoxiclav was discontinued.

Subsequent MRI head showed new ring-enhancing lesions within the temporal lobe and post-central gyrus. MRI of his right forearm showed a 30x15x15 mm septated phlegmon replacing the pronated quadratus. PET-CT showed left apical consolidation, multiple areas of muscular uptake and scattered ring-enhancing intra-cranial lesions. Ceftriaxone was substituted to co-trimoxazole to cover Nocardia and Toxoplasma.

Blood cultures, HIV test, urinary legionella, pneumococcal, histoplasma and serum cryptococcal antigens were negative. Serum beta-D-glucan was >500pg/ml and galactomannan negative. A lumbar puncture revealed a normal CSF white count, protein and glucose with negative beta-D-glucan and cryptococcal antigen.

He responded well to antimicrobials with defeverscence and falling CRP.

After stabilisation, he elected to return to Australia for further management.

A biopsy of his right forearm mass demonstrated beaded, branching Gram-positive bacilli on microscopy, consistent with Nocardia (provisional speciation *N. Brasiliensis*).

**Discussion:**

This case posed multiple diagnostic challenges. At presentation, the differential diagnoses were broad and included metastatic cancer with paraneoplastic fasciitis, opportunistic infection, atypical multisystem sarcoidosis or dual pathology. As investigations were resulted, an infective cause was suspected and ultimately, Nocardia was diagnosed.

Nocardia are aerobic, branching Gram-positive bacilli, that are ubiquitous in the environment and predominantly found in soil, water and decaying matter. Infection can occur through inhalation, or direct inoculation.

Disseminated Nocardiosis is traditionally regarded as an opportunistic infection. Predisposing factors typically affect cell-mediated immunity and include corticosteroids, HIV infection, certain chemotherapeutic regimes and immunosuppression in autoimmune diseases, solid organ transplantation or haemopoietic stem cell transplant.

Nocardiosis is a relatively rare infection though incidence is increasing globally. Certain regions of Australia, including Queensland, our patient’s state of residence, are recognised as having a particularly high incidence. Clinical suspicion for Nocardial infection in immunocompromised patients should be raised in those presenting with indolent or insidious symptoms particularly involving the brain and lung simultaneously; importantly systemic features such as fever maybe absent.

Diagnosis can be difficult as the bacteria are slow growing in the laboratory, taking two-three weeks to culture. In this case, tissue diagnosis was hampered by the patient’s wish to return to Australia to complete investigations.

Treatment involves prolonged antibiotic courses: initial empiric intravenous therapy with co-trimoxazole, aminoglycosides or carbapenems with subsequent oral stepdown. Reduction in immunosuppressive therapy should be considered. Even with appropriate management, Nocardiosis is associated with a high mortality, ranging from 16-32%, with estimates of up to 80% when there is brain or spinal cord involvement. Nocardiosis can relapse, with relapse rates of up to 5% in patients with solid organ transplantation. Future treatment decisions regarding antimicrobial prophylaxis and ongoing immunosuppressive management, make this case challenging.

**Key learning points:**

• Corticosteroids exert multiple immunosuppressive effects, including inhibition of the transcription pro-inflammatory cytokines and decreased function of leukocytes. They increase the risk of serious bacterial infection, in a dose-dependent manner. Corticosteroids also predispose to the development of opportunistic infections, including Pneumocystis Jirovecii, atypical mycobacteria, fungal infections, cytomegalovirus, endemic mycoses, cryptococcus, aspergillus and disseminated candidiasis. The risk of opportunistic infection is highest in the first year of treatment. It is important for clinicians to be alert to the possibility of opportunistic infections in managing immunocompromised patients who are unwell and to therefore maintain a broad list of differential diagnoses in such scenarios. A multi-disciplinary management approach including early consultation with infectious diseases is vital to ensure accurate diagnosis, timely treatment and positive outcomes for our patients.

• Nocardiosis is a relatively uncommon bacterial pathogen that is traditionally considered an opportunistic infection. The disease can involve the lungs, brain, skin or soft tissues. Diagnosis can be challenging due to heterogenous clinical manifestations and difficulty in bacterial culture. Biopsy is needed for definitive diagnosis.

• There is marked geographical variation in the prevalence and species of Nocardia. Nocardiosis is rare in the UK, with one case series identifying 18 cases over an 18-year period at a large tertiary hospital in London. In keeping with our case report, Nocardia is more common in Australia with one case series describing 484 cases in seven major Queensland Hospitals over a 15-year time period.

• Although a rare infection, an awareness of Nocardiosis is important to UK rheumatologists due to its increasing global prevalence and associated high mortality, especially if diagnosis is delayed. Prolonged antimicrobial treatment is required with step down to long term prophylaxis if immunosuppression cannot be discontinued.

